# Immunogenicity, safety, and efficacy of sequential immunizations with an SIV-based IDLV expressing CH505 Envs

**DOI:** 10.1038/s41541-020-00252-w

**Published:** 2020-11-18

**Authors:** Maria Blasi, Donatella Negri, Kevin O. Saunders, Erich J. Baker, Hannah Stadtler, Celia LaBranche, Benjamin Mildenberg, Georgeanna Morton, Andrew Ciarla, Xiaoying Shen, Yunfei Wang, Wes Rountree, Bala Balakumaran, Sampa Santra, Barton F. Haynes, Anthony M. Moody, Andrea Cara, Mary E. Klotman

**Affiliations:** 1 Department of Medicine, Duke University School of Medicine, Durham, NC USA; 2 Duke Human Vaccine Institute, Duke University School of Medicine, Durham, NC USA; 3 Department of Infectious Diseases, Istituto Superiore di Sanità, Rome, Italy; 4 Department of Surgery, Duke University School of Medicine, Durham, NC USA; 5 Beth Israel Deaconess Medical Center, Boston, MA USA; 6 Department of Pediatrics, Duke University School of Medicine, Durham, NC USA; 7 National Center for Global Health, Istituto Superiore di Sanità, Rome, Italy; 8 Present Address: Harvard Law School, Cambridge, MA USA

**Keywords:** Vaccines, HIV infections, Immunology

## Abstract

A preventative HIV-1 vaccine is an essential intervention needed to halt the HIV-1 pandemic. Neutralizing antibodies protect against HIV-1 infection in animal models, and thus an approach toward a protective HIV-1 vaccine is to induce broadly cross-reactive neutralizing antibodies (bnAbs). One strategy to achieve this goal is to define envelope (Env) evolution that drives bnAb development in infection and to recreate those events by vaccination. In this study, we report the immunogenicity, safety, and efficacy in rhesus macaques of an SIV-based integrase defective lentiviral vector (IDLV) expressing sequential gp140 Env immunogens derived from the CH505 HIV-1-infected individual who made the CH103 and CH235 bnAb lineages. Immunization with IDLV expressing sequential CH505 Envs induced higher magnitude and more durable binding and neutralizing antibody responses compared to protein or DNA +/− protein immunizations using the same sequential envelopes. Compared to monkeys immunized with a vector expressing Envs alone, those immunized with the combination of IDLV expressing Env and CH505 Env protein demonstrated improved durability of antibody responses at six months after the last immunization as well as lower peak viremia and better virus control following autologous SHIV-CH505 challenge. There was no evidence of vector mobilization or recombination in the immunized and challenged monkeys. Although the tested vaccines failed to induce bnAbs and to mediate significant protection following SHIV-challenge, our results show that IDLV proved safe and successful at inducing higher titer and more durable immune responses compared to other vaccine platforms.

## Introduction

Vaccine-induced immunity is generally provided by the elicitation of protective and durable antibody responses. The HIV-1 envelope (Env) is the only target of neutralizing antibodies (nAb)^1^. Several strategies have been evaluated to induce broadly neutralizing antibodies (bnAbs), including the use of various immunization strategies and Env-based immunogens. However, none of these strategies have been effective at inducing broadly bnAbs in humans or in non-human primates (NHPs)^2–4^. Antibody-virus co-evolution studies performed on HIV-1 infected individuals from the time of transmission to bnAb development have demonstrated that bnAbs arise after extensive Env diversification^5–7^. Thus, one strategy to induce bnAbs is to perform sequential immunizations with a series of Env isolates from an individual that made bnAbs to mimic natural infection by vaccination and guide bnAb development.

The other crucial aspect of a successful vaccine strategy is the selection of an antigen delivery system that can provide high magnitude and durable immune responses. In the modestly effective RV144 trial^8^, the estimated vaccine efficacy dropped significantly between 6 months to 12 months after vaccination, which correlated with the rapid decline of vaccine-induced antibody responses highlighting the importance of maintaining adequate concentrations of protective antibodies (Abs) over time.

We have shown, in both mice and macaque studies, that integrase defective lentiviral vectors (IDLV) provide prolonged antigen expression and can induce potent and durable antigen-specific immune responses^9–13^. We demonstrated that immunization with an SIV-based IDLV expressing an HIV-1 Env induces continued antibody affinity maturation up to three months post-prime, which is further improved upon additional IDLV-Env immunizations^12^. Furthermore we demonstrated persistent antigen expression at the site of injection up to three months post intramuscular immunization^12,14^, suggesting a direct correlation between continuous antigen expression by IDLV and durability of antigen-specific immune responses^14^.

The SIV-based IDLV has been engineered with a number of features to enhance its safety, including the deletion of the promoter/enhancer elements present in the U3 region of the LTR^15^. This self-inactivating (SIN) feature, together with the episomal configuration of IDLV, markedly reduces the risk of vector recombination that could result in production of replication-competent lentivirus (RCL), insertional mutagenesis, and mobilization of the vector from transduced target cells by subsequent infection with a replication-competent virus. However, the possibility of recombination between IDLV and a replication-competent virus has never been tested in NHPs.

Here we report that repeated sequential immunization with IDLV expressing gp140 Env immunogens derived from the CH505 HIV-1 infected individual who made the CH103 and CH235 CD4 binding site bnAb lineages^5,7,16^, was immunogenic and safe in rhesus macaques. We demonstrate that IDLV-CH505-Env +/− CH505 Env protein-induced higher magnitude and durability of antibody responses compared to protein alone, DNA and DNA + protein immunization strategies using the same Env immunogens. Following repeated low-dose intrarectal challenges with the autologous SHIV.CH505.375H.dCT^17,18^ virus, one out of eight animals in the IDLV + protein group resisted infection and this group of animals had 3.6 times lower peak viral load compared to the control group of animals immunized with an IDLV expressing GFP (*n* = 11). Differently from animals immunized with IDLV-CH505 Env alone and animals in the control group, we observed rapid and sustained viremia control in the IDLV + protein group that correlated with the magnitude and quality of the antibody responses. Finally, to evaluate the safety of sequential IDLV-CH505 Env immunization a group of four vaccinated animals was challenged intravenously with SHIV.CH505.375H.dCT to enhance the possibility of potential recombination and/or mobilization events between the challenge virus and the IDLV vaccine. No recombination/mobilization events were detected in these animals. Our study demonstrates that IDLV is a safe and effective vaccine platform that induces high magnitude and durable immune responses against HIV-1.

## Results

### Study design

**Fig. 1:**
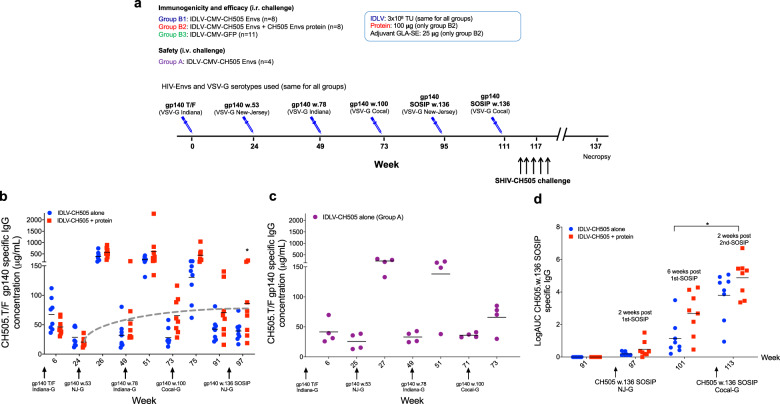
Magnitude and durability of antibody responses induced by sequential IDLV-CH505 Env immunizations. **a** Non-human primate immunization regimen with sequential IDLV-CH505 Env +/− protein. The dose of IDLV, protein and adjuvant used for each immunization as well as the challenge and necropsy schedules are indicated. A group of eleven animals were sequentially immunized with IDLV-GFP as the control arm. The HIV-Envs encoded by IDLV are the same for all the vaccine groups (A, B1, and B2) and the VSV-G serotypes used for vector pseudotyping are the same for all the groups. **b** ELISA binding of plasma antibodies to CH505 T/F Env at the peak and six months post-each immunization with IDLV-CH505 (group B1 animals) and IDLV-CH505 + protein (group B2 animals). Binding titers measured as concentration in µg/mL starting at a 1:3000 plasma dilution. The dotted gray line indicates the improved durability of antibody responses at six months post-each IDLV-CH505 immunization. Asterisks indicate that a statistically significant improvement in the magnitude of Ab responses was detected between weeks 24 and 97 (*p* = 0.0234). **c** ELISA binding of plasma antibodies to CH505 T/F Env at the peak and six months post-each immunization with IDLV-CH505 (group A animals, safety arm). **d** ELISA binding of plasma antibodies to the CH505w.136 SOSIP trimer of plasma samples collected after immunization with IDLV-CH505w.136 SOSIP. Binding titers measured as Log area under the curve (Log AUC) starting at a 1:30 plasma dilution. No envelope binding was detected in plasma samples collected before the first immunization. Asterisks indicate that a statistically significant improvement in the magnitude of Ab responses was detected between weeks 101 and 113 (*p* = 0.0156).

The set of CH505 Envs used in this study were selected based on binding to stages of the CH103 bnAb lineage: the CH505 transmitted founder (T/F) and three natural CH505 variants (week 53, 78, and 100)^16^. Thirty-one Indian rhesus macaques were divided into four immunization groups as shown in Fig. 1a; one group of eight animals was immunized intramuscularly with IDLV expressing sequentially evolved CH505 gp140 Envs (T/F, w53, w78, w100) at six months intervals (Group B1—IDLV-CH505 alone). The second group of eight animals was immunized intramuscularly with IDLV expressing sequentially evolved CH505 Envs in combination with the corresponding CH505-gp140 protein in GLA-SE^19^ adjuvant (Group B2—IDLV-CH505 + protein). For the last two immunizations of both B1 and B2 group animals, we used IDLV expressing a stabilized gp140 SOSIP Env trimer (CH505.w136 SOSIP) with the goal of focusing antibody responses towards the epitopes targeted by different bnAbs^4,20,21^. A third group of 11 animals served as the control group and received sequential immunizations with IDLV-GFP (Group B3). Six weeks after the last immunization, monkeys in groups B1, B2, and B3 received ten intrarectal challenges with SHIV.CH505.375H.dCT, whereas, a fourth group of four animals, also immunized intramuscularly with IDLV expressing sequentially evolved CH505 Envs (Group A), was challenged once intravenously to maximize the chances of possible recombination between IDLV and the replication-competent SHIV-CH505, as assessed in the safety studies described below.

### Improved durability of Env-specific Ab responses post sequential IDLV-Env immunization

Plasma Abs specific for CH505.T/F-Env were assessed at two weeks post each immunization and then monthly thereafter. All NHPs developed high titers of CH505.T/F-gp140 Env-specific Abs at 6 weeks post-prime (Fig. 1b), which were strongly boosted after each subsequent IDLV-CH505 Env immunizations. Immunizations with IDLV-CH505 alone elicited similar antibody responses as IDLV-CH505 + protein at the prime, however after the subsequent boosts antibody titers were higher in the IDLV-CH505 + protein co-immunization group (Fig. 1b). In this group of animals, antibody titers at 6 months after the IDLV-CH505.w.100 gp140 immunization (week 97) were significantly higher (*p* = 0.0234 based on a Wilcoxon signed-rank test) than those detected at 6 months after the first immunization (week 24) (Fig. 1b), suggesting that the durability and magnitude of the vaccine-induced antibody response improved over time. A similar trend was also observed in group B1 animals, although the peak antibody titers after the 4th immunization with IDLV-CH505.w.100 gp140 was lower compared to the previous immunization. Similar antibody titers as in group B1 animals were induced in the four animals in group A, also immunized intramuscularly with IDLV-CH505 alone (Fig. 1c).

We observed that following the fifth immunization with IDLV expressing the CH505.w136 SOSIP, there was no boost in the antibody response induced by the previous immunization with non-stabilized gp140 Envs, suggesting that IDLV-CH505.w136 SOSIP immunization induced Abs against epitopes only present on the closed Env trimer. To confirm this, we performed PGT151 SOSIP capture ELISAs to detect anti-CH505.w136 SOSIP specific antibodies. As shown in Fig. 1d, anti-CH505.w136 SOSIP specific Abs were elicited in all animals between 2 and 6 weeks post-vaccination and were significantly boosted (*p* = 0.0156 for both comparisons using Wilcoxon signed-rank tests) by a second IDLV-CH505.w136 SOSIP +/− protein immunization. These data suggest that immunization with IDLV-CH505.w136 SOSIP induced a narrower and more specific Ab response compared to immunizations with non-stabilized gp140 Envs.

### Epitope mapping of antibody response induced by sequential IDLV-CH505 immunizations

**Fig. 2:**
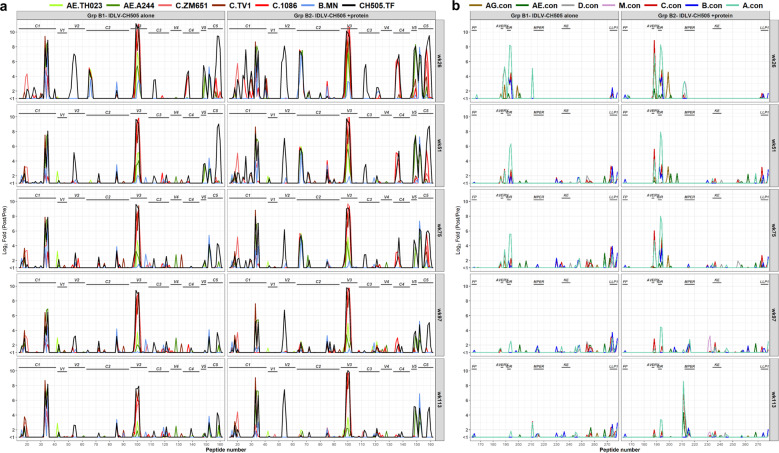
Linear epitope specificities of antibody responses induced by sequential IDLV-CH505 Env immunizations. gp120 (**a**) and gp41 (**b**) binding plots for serum samples from macaques immunized with either IDLV-CH505 alone or IDLV-CH505 + protein at 2 weeks after each immunization. Mean binding responses for each of the vaccine groups are shown. Numbers on the *x* axis are peptide numbers in the array library. The number on the *y* axis indicates the magnitude of binding calculated as the log2 fold difference, post-/pre-immunization intensity. Different colors of bars represent different strains/clades as indicated in the keys in the panels (TH023, A244, ZM651, TV-1, 1086C, MN, CH505 for panel a and AG, AE, D, M, C, B and A consensus for (**b**)).

**Fig. 3:**
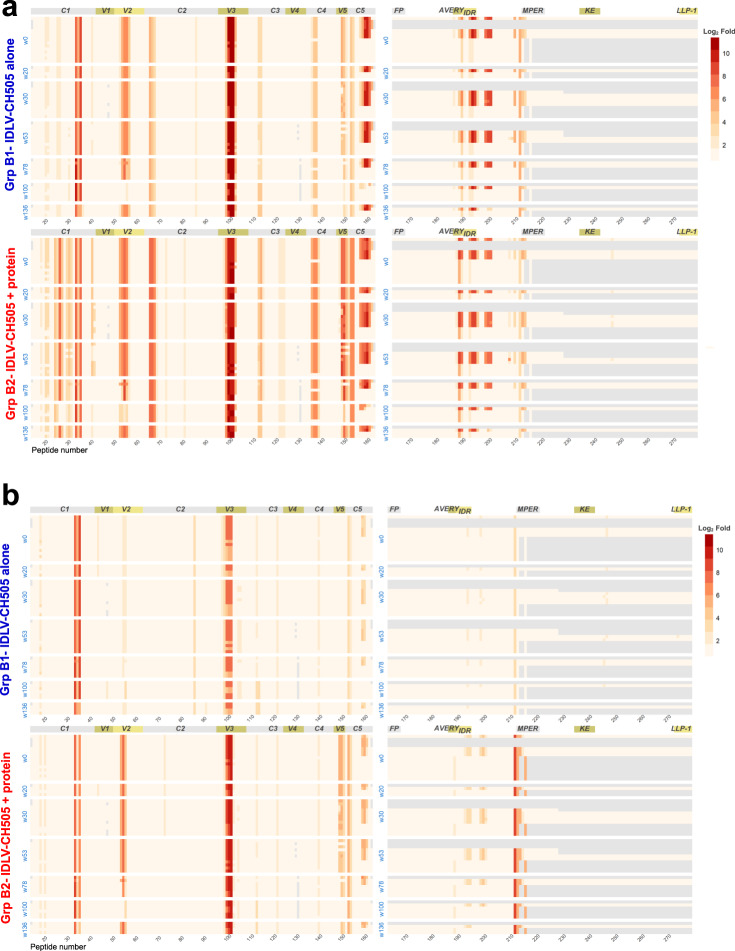
Linear epitope binding to CH505 sequences. The heat maps show gp120 and gp41 binding, at week 26 (**a**) and week 113 (**b**) post IDLV +/− protein immunization, to the different CH505 strain sequences used in immunization. Binding intensity is shown for each peptide, corrected with its own background value. Light gray shaded areas indicate sequence not present in the array library.

We next characterized the epitope specificities of the Abs induced by the sequential IDLV-CH505 Envs +/− protein immunizations by peptide microarray. Serum samples collected at 2 weeks after each boost immunization (weeks 26, 51, 75, 97, and 113) were tested for their ability to bind to a panel of Env sequences from different clades. Immunized animals in both groups developed cross-clade responses against a range of linear epitopes in gp120 (Fig. 2a) and gp41 (Fig. 2b), including C1, V3, V5, C5, AVERY, gp41 IDR, and MPER. Binding against the variable loop 2 (V2) was also detected in all the animals but was primarily specific for CH505 sequences (Fig. 2a). Binding specificities were overall similar between the two groups (Supplementary Fig. 1), but the magnitude of the responses trended higher in group B2 animals for the majority of the epitopes (Fig. 2). Following IDLV-CH505.w136 SOSIP boosts a reduction in the magnitude of binding responses against different epitopes was observed in both groups of animals (Fig. 3). Differences were observed for V2 and MPER binding responses between group B1 and B2 animals after the last IDLV- CH505.w136 SOSIP immunization (Fig. 3b). These data further suggest that immunization with IDLV-CH505.w136 SOSIP induced a narrower Ab response compared to immunizations with non-stabilized gp140 Envs and that the addition of the CH505.w136 SOSIP protein in group B2 animals resulted in a higher magnitude of antibody binding against specific epitopes, including V2 and MPER.

### Sequential IDLV-CH505 immunizations induce high-titers of tier-1 nAb responses but no breadth

We next assessed the serum neutralizing activity induced by sequential IDLV-CH505 Envs immunization. Neutralization assays were performed on serum samples collected at different time points post-immunization including at the peak of the antibody response (weeks 6, 26, 51, 75, 101, and 113), 6 months after the second (week 49) and fourth (week 91) immunizations as well as 2, 11, and 16 weeks (weeks 97, 106, and 111 respectively) after the first IDLV-CH505.w136 SOSIP immunization and 2 weeks (week 113) after the second IDLV-CH505.w136 SOSIP immunization. Serum samples from all 16 macaques in groups B1 and B2 neutralized the autologous tier 1 virus CH505 w4.3 after the second immunization with IDLV-CH505.w53 gp140 Env +/− protein (Table 1). The CH505w4.3 tier-1 virus is a natural variant that was isolated from the CH505 individual at week 4 post-infection. It has a single amino acid mutation compared to the tier 2 T/F virus (W to G in the MPER region) that is responsible for the tier 1 phenotype^7^. Significantly higher tier 1 nAb titers were detected in group B2 animals (IDLV-CH505 + protein) compared to group B1 animals (IDLV-CH505 alone) across all time points (*p* < 0.0001 using an aligned rank test). An increase in nAb titers was detected after each immunization except after the last IDLV-CH505.w136 SOSIP boost. Despite the strong tier 1 nAb response, neutralization of tier 2 viruses was not detected: titers against the four tier 2 sequential autologous viruses (CH505 TF, week 53, week 78, week 100) and the tier 2 challenge SHIV CH505.375H were all negative. To determine whether CD4bs bnAb antibody lineages were being stimulated by these immunizations, we assayed viruses that are sensitive to neutralization by the inferred unmutated common ancestor and intermediate Abs in the VRC01, CH235, and CH103 lineages^22^ with sera starting at week 51 (post 4th immunization). We were unable to detect the neutralization of any of these viruses. The absence of bnAb precursors or tier 2 nAb indicates a lack of the potential for broadly nAb despite elicitation of high titer tier 1 nAb by sequential IDLV-CH505 Env immunizations.

**Table 1:** Serum neutralization activity against the clade C tier1 virus CH505.w4.3.

Immunogen →		T/F	w.53		w.78	w.100		w.136 SOSIP			w.136 SOSIP	
Vaccine Group	Animal ID	Week 6	Week 26	Week 49	Week 51	Week 75	Week 91	Week 97	Week 101	Week 106	Week 111	Week 113
Group B1 IDLV-CH505 alone	6597	<20	8199	113	3715	597	96	1298	4083	1502	117	<20
	6599	<20	356	<20	341	101	28	60	579	106	37	82
	6595	<20	471	<20	990	892	<20	921	1610	366	478	21
	6575	<20	2970	240	203	878	60	58	<20	<20	26	64
	6592	<20	810	153	215	125	74	67	223	84	56	44
	6596	<20	188	<20	106	127	175	239	497	74	68	63
	6586	<20	872	585	1285	1317	1162	824	488	405	130	336
	6589	<20	2629	317	521	814	769	142	337	262	<20	34
Group B2 IDLV-CH505 + protein	6582	21	3034	463	3200	7611	1228	6056	8083	10302	2605	2030
	6600	<20	752	252	8110	7104	1748	6570	7382	2814	503	587
	6601	<20	4860	366	152,43	23,098	718	18961	7802	2170	316	1095
	6578	<20	385	<20	43	97	23	57	64	<20	<20	32
	6603	<20	3876	534	2706	4676	1026	2945	2447	1254	544	897
	6598	<20	9908	540	6444	11,059	486	4721	6691	2041	395	297
	6577	<20	8873	<20	26	900	<20	<20	170	23	<20	76
	6584	<20	2819	36	1459	631	49	60	135	52	20	87

Values are the serum dilutions at which relative luminescence units (RLU) were reduced by 50% compared to RLU in virus control wells after subtraction of background RLU in cell control wells. A response was considered positive if the post-immunization ID50 was three times higher than the pre-immune ID50 and three times greater than the signal against the MLV-pseudotyped negative control virus. No neutralization against other CH505 tier 1 and tier 2 viruses (CH505.T/F (tier 2), CH505.w53.16 (tier 1a), CH505.w.78 (tier 1b) and CH505.w.100 (tier 2)) was detected.

### Sequential IDLV-CH505 immunizations induce higher binding and nAb titers than protein and DNA +/− protein immunization regimens

**Fig. 4:**
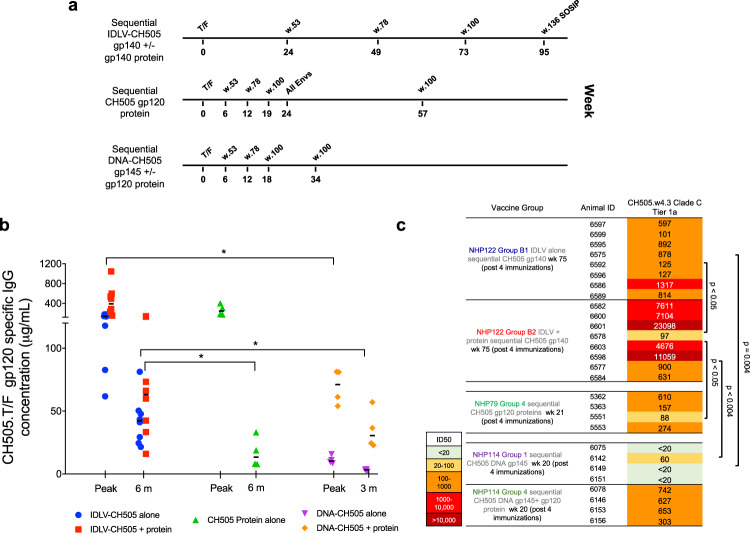
Magnitude and durability of antibody responses induced by sequential IDLV, protein, and DNA +/− protein immunizations. **a** Non-human primate immunization regimens with sequential IDLV +/− protein (NHP122 study), protein alone (NHP79 study) and DNA +/− protein (NHP114 study). **b** ELISA binding of plasma antibodies to CH505 T/F Env at the peak and either 6 or 3 months (for DNA +/− protein) post four immunizations with each vaccine regimen. Binding titers measured as concentration in µg/mL starting at a 1:3000 plasma dilution. Asterisks indicate that a statistically significant difference was detected (*p* = 0.004 for IDLV-CH505 + protein vs DNA +/− protein and IDLV-CH505 alone vs DNA alone at peak; *p* = 0.0162 for IDLV-CH505 alone vs DNA + protein at peak; *p* = 0.0283 for IDLV-CH505 alone vs CH505 protein alone at 6 months; *p* = 0.0162 for IDLV + protein vs CH505 protein alone at 6 months; *p* = 0.004 for IDLV-CH505 +/− protein vs DNA alone at the 3 vs 6 months comparison). **c** Serum neutralization activity against the clade C tier1 virus CH505.w4.3 post four immunizations with each vaccine regimen.

We next compared the magnitude and durability of binding and nAb responses between sequential IDLV +/− protein, protein alone and DNA +/− protein immunization regimens delivering the same sequential CH505 Envs^23,24^. The same adjuvant (GLA-SE) was used in all the regimens that included protein. The immunization schedule for each of the vaccine regimens tested is shown in Fig. 4a. Of note, while in the IDLV-based regimens the animals were immunized every 6 months, in the protein and DNA +/− protein regimens the animals were immunized every 6 weeks before receiving a delayed boost at either 4 or 8 months, respectively. Significantly higher magnitude of antibody responses were induced in the IDLV +/− protein groups compared to DNA +/− protein groups (Fig. 4b). The durability of antibody responses at six months post-vaccination was also significantly higher in the IDLV +/− protein groups compared to 6 months post- protein or 4 months post DNA alone. Similarly, nAb titers were significantly higher in the IDLV +/− protein groups compared to DNA alone and in the IDLV + protein group compared to protein (Fig. 4c). Although the median nAb titer values for the IDLV + protein group was 5890 and for the DNA + protein group was 640, this difference did not reach statistical significance due to the small sample size of the DNA + protein group (*n* = 4). These comparisons demonstrate that IDLV +/− protein induced a more durable antibody response compared to protein alone and DNA +/− protein vaccine regimens.

### Efficacy of sequential IDLV-CH505 vaccination against autologous SHIV challenge

**Fig. 5:**
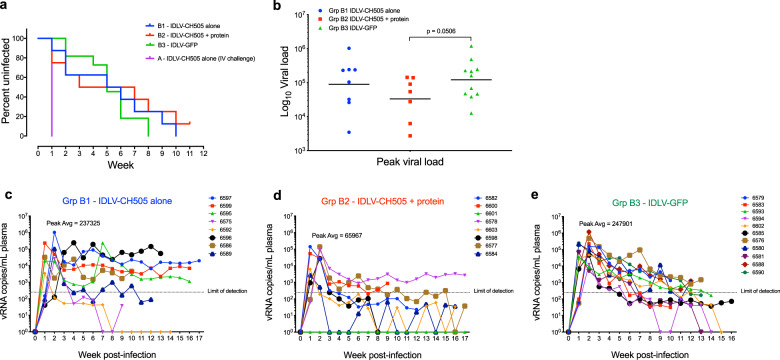
SHIV challenge outcomes in IDLV-CH505 +/− protein vaccinated animals. **a** Six weeks after completion of the scheduled vaccinations (week 117) the 16 macaques in the B1 and B2 vaccine groups and the 11 macaques in the B3 control group were challenged intrarectally with the Clade C tier 2 SHIV.CH505.375H.dCT. The four animals in group A were challenged once intravenously. The Kaplan–Meier plot shows the percentage of uninfected macaques after 10 weekly IR challenges in each group. **b** Peak viral load of the infected macaques from the vaccine and control groups. Lines indicate geometric means (*p* = 0.0506, Exact-Wilcoxon test). **c**–**e** Viral load trends from time to infection in each group. Each line represents one animal.

To assess the efficacy of the sequential IDLV +/− protein vaccines, we challenged animals in B1, B2, and B3 groups beginning at week 117 (6 weeks after the last immunization) ten times with a weekly intrarectal low-dose autologous Tier-2 SHIV.CH505.375H.dCT. Eleven animals immunized with an IDLV-GFP vector were challenged as the control arm. After ten challenges, only one of the eight IDLV-CH505 + protein vaccinated animals remained uninfected, while all the animals in the IDLV-CH505 group became infected. In the control arm (IDLV-GFP) all the animals became infected after 8 challenges (Fig. 5a). There was a difference between IDLV-CH505 + protein, IDLV-CH505 alone and the IDLV-GFP control groups in peak viral load, with the IDLV-CH505 + protein group having ~3.6 times lower peak viral load compared to IDLV-CH505 alone or IDLV-GFP (Fig. 5b). Furthermore, we observed more rapid and sustained control of viremia in the IDLV-CH505 + protein group compared to IDLV-CH505 alone group, with 71% (5/7) of group B2 animals and 37.5% (3/8) of group B1 animals having viral loads below the limit of detection at the time of necropsy (10 weeks post last challenge) (Fig. 5c, d). Virus control was observed also in 45.4% (5/11) of the animals in the control arm (IDLV-GFP) (Fig. 5e).

Although our study did not include a naive group of animals, historical data on unvaccinated animals challenged with the same SHIV used in our study have been published recently^18^. Similarly to our study, animals were challenged intrarectally with a 1:100 dilution of SHIV.CH505.375H.dCT challenge stock (the same stock used in our study) and VLs of individual animals were assessed up to 16 weeks post-challenge^18^. All the animals in that study became infected after 5 IR challenges and the VLs were sustained over time after the peak post-infection. On the contrary, in our study it required eight challenges for all the animals in the IDLV-GFP group to become infected and VLs declined over time, suggesting that the innate and/or Gag-specific immune responses induced by IDLV-GFP injection had an impact on the timing of virus acquisition and VL levels.

**Fig. 6:**
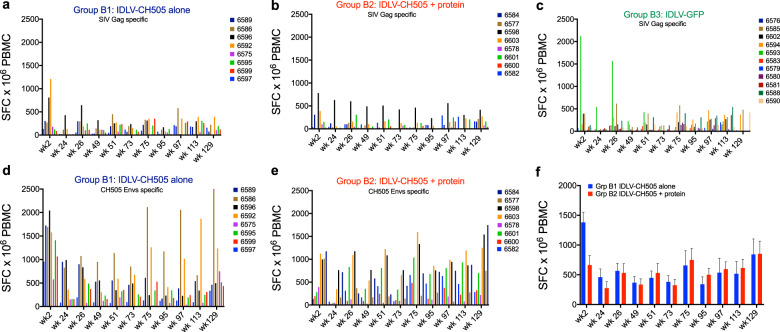
Env and Gag-specific T-cell responses induced by IDLV-CH505 +/− protein and IDLV-GFP immunizations. **a**–**c** SIV-Gag and **d**–**e** CH505 Envs specific T cell responses were measured by IFN-γ ELISPOT in both the IDLV-CH505 +/− protein and IDLV-GFP vaccinated animals. Results are expressed as IFN-γ secreting cells, measured as spot forming cells (SFC) per million PBMC, at different time points post vaccination. Each line represents one animal. **f** Group means IFN-γ ELISPOT anti-Env responses over time. Error bars indicate S.E.M.

We recently demonstrated that therapeutic vaccination of chronically SHIV-infected macaques with an IDLV expressing SIV-Gag, resulted in durable viremia control^25^. Because SIV-Gag is present in both the IDLV-CH505 and IDLV-GFP vector particles, we next measured anti SIV-Gag-specific T cell responses in the three groups of animals to determine whether differences in SIV-Gag responses between animals that controlled or did not control viral load could be detected. Similar anti SIV-Gag responses were detected in the three groups of animals before and after the challenge (Fig. 6a–c). The anti-CH505 Env T cell responses were also similar between group B1 (IDLV-CH505 alone) and group B2 (IDLV-CH505 + protein) animals (Fig. 6d–f). No correlation between IFN-γ responses (weeks 75, 97, and 101) and time to virus acquisition was observed. These data suggest that the vaccine-induced T cell responses did not influence the time to infection.

As some of the vaccinated animals in group B1 showed sustained viral load levels after infection, we analyzed the data from neutralization assays against the SHIV CH505.375H challenge virus at week 113 (prior to challenge) for enhancement of virus infection in vitro but detected none. Given that all the animals in this study received the same number of vector immunizations, this phenomenon is not due to IDLV immunization.

### Binding and nAb titers correlate with time to virus acquisition

**Fig. 7:**
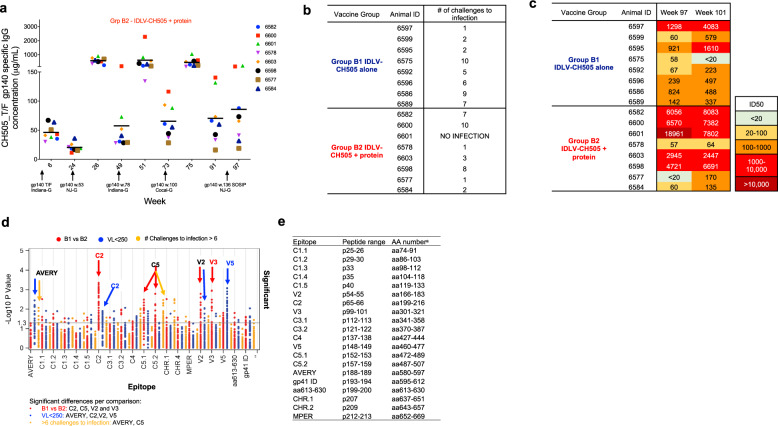
Magnitude and specificity of antibody responses in IDLV + protein vaccinated animals correlate with time to virus acquisition. **a** ELISA binding of plasma antibodies to CH505 T/F Env at the peak and six months post-each immunization with IDLV-CH505 + protein (group B2 animals). Each dot represents one animal. **b** Number of challenges to virus acquisition for each animal in groups B1 and B2. **c** Serum neutralization activity against the clade C tier1 virus CH505.w4.3 at the indicated time points. **d** Comparison of linear epitope specificities between animals in B1 and B2 vaccine groups (red dots), animals with viral loads above or below 250 copies/mL (blue dots) and animals that required either <6 or >6 challenges to become infected (yellow dots). Significant differences fall above the horizontal line and are listed in the text box below the figure for each comparison. **e** The peptide range and the aa position for each epitope are indicated.

To determine whether the differences in binding and nAb titers detected between the two vaccine groups played a role in the observed challenge outcome, we performed Kendall Tau correlation analyses. A correlation between binding antibody titers at weeks 75 and 97 (Fig. 7a) and time to virus acquisition (Fig. 7b) was observed (Kendall tau correlation *p* < 0.05) for group B2 animals (IDLV-CH505 + protein). The animal that remained uninfected (6601) and the one that was infected at the last challenge (6600) demonstrated the higher magnitude of binding antibody responses (Fig. 7a), while the animals that got infected between 1 and 2 challenges (6577, 6578, 6584) had the lowest antibody titers. Similarly, a correlation between nAb titers at weeks 101 for group B1 and weeks 75, 97, and 101 for group B2 (Fig. 7c) and time to virus acquisition was observed (Kendall tau correlation *p* < 0.05).

Tier-1 nAb titers increased significantly post-challenge in all the animals, except in the one animal that remained uninfected (6601), while very low tier-2 nAb titers were detected in a few of the animals (Supplementary Table 1).

Because we observed quantitative differences in epitope binding specificities between the two vaccine regimens (Fig. 3), we next compared epitope binding responses among animals based on vaccine group (B1 vs B2), viral load (below and above 250 copies/mL) and time to acquisition (higher and lower than six challenges to infection). We observed significant differences (Exact-Wilcoxon test) in epitope binding responses between group B1 and B2 for C2, C5, V2, and V3 epitopes (Fig. 7d–e). When comparing epitope binding specificities between controller vs non controller animals, significant differences in binding to AVERY, C2, V2, and V5 epitopes were observed. In addition, the animals that required more than six challenges to infection showed higher binding to AVERY and C5 epitopes.

These data suggest that the differences in nAb titers and the magnitude of Ab specific for certain epitopes between the two vaccine regimens played a role in the challenge outcome.

### Absence of mobilization and or recombination between IDLV and the challenge virus

**Fig. 8:**
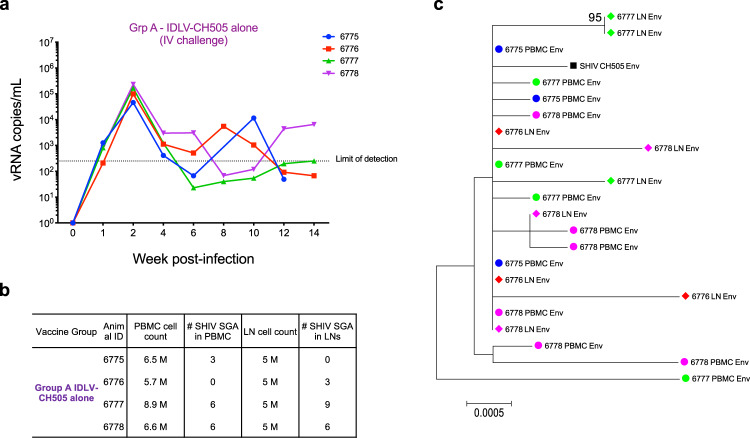
Absence of vector mobilization or recombination in IDLV-CH505 vaccinated animals challenged intravenously with SHIV-CH505. **a** Viral load trends from time of infection in group A animals (IDLV-CH505 alone). Each line represents one animal. **b** Number of SHIV.CH505.375H.dCT sequences amplified from either PBMC or lymph nodes samples for each animal. **c** Neighbor-joining phylogenetic tree including CH505 env sequences amplified from either PBMC (full circles) or lymph nodes (full diamonds) from each animal. Each color represents one animal. Bootstrap values ≥80 are shown. Genetic distance is indicated at the bottom of the figure and represents the number of nucleotide substitutions per site.

To evaluate the safety of sequential IDLV-CH505 Env immunization a group of 4 macaques was challenged intravenously with SHIV.CH505.375H.dCT (Fig. 1a) to enhance the possibility of potential recombination and/or mobilization events between the challenge virus and the IDLV-CH505 vaccines. All the animals became infected after one IV challenge (Fig. 5a). The mean peak plasma viral load for the group was 136,322 copies/mL and at the time of necropsy (12 weeks post-challenge) two of the four animals had viral loads below 250 copies/mL (Fig. 8a). To assess the possibility of recombination and/or mobilization events, we performed single genome amplification (SGA) on peripheral blood mononuclear cells (PBMC) and lymph node cells using primer sets designed to amplify any of the five CH505 gp140 *env* sequences encoded by IDLV (Supplementary Fig. 2). We could not amplify any vector sequence in either PBMC or lymph node cells. Conversely, using SHIV.CH505.375H.dCT specific primers we were able to amplify several SHIV sequences in both PBMC and LN cells (Fig. 8b, c). These data demonstrate no tendency for IDLV to recombine with actively replicating virus in an infected host and indicate that IDLV is a safe platform for HIV-1 vaccine that can be used in repeated immunizations.

## Discussion

In this study, we evaluated the immunogenicity, safety, and efficacy of sequential immunizations of rhesus macaques with an SIV-based IDLV, with or without protein, expressing a series of Envs isolated from the CH505 individual who made the CH103 and CH235 broadly neutralizing antibody (bnAb) lineages. Our results show that co-immunization with IDLV and protein-induced higher magnitude and durability of antibody responses compared to IDLV alone. In the IDLV + protein co-immunized animals, we detected higher titers of nAb and higher V2 and MPER epitope binding after the last IDLV-CH505.w136 SOSIP immunization, suggesting that the addition of the protein improved neutralizing Abs titers and the magnitude of Abs against specific epitopes in the vaccinated macaques.

We also compared the magnitude and durability of the antibody response induced by IDLV-CH505 +/− protein vaccination to those induced by protein and DNA +/− protein vaccine regimens delivering the same series of CH505 Envs in historical NHP studies^23,24^. Our analysis showed that co-immunization with IDLV and protein was superior to any of the tested vaccine regimens in terms of magnitude and durability of the immune responses at 6 months post-vaccination. Furthermore, IDLV alone induced higher magnitude of antibody responses compared to DNA +/− protein and more durable antibody responses compared to protein alone or DNA +/− protein. Similarly, serum neutralizing activity was significantly higher in the IDLV + protein co-immunized animals compared to all the other tested vaccine regimens. These data support the use of IDLV as a vaccine platform to induce higher magnitude and more durable immune responses against HIV-1 as well as other pathogens.

Consistent with previous studies that performed sequential immunizations of macaques with gp120 or gp140 CH505 Envs^23,24^, we did not detect neutralization breadth in the sera of the vaccinated animals in this study. Several studies have shown that SOSIP trimers engineered for increased Env stability better mimic native, virion-associated trimers both antigenically and structurally, including by displaying the epitopes for many bnAbs^4,20,21,26,27^. We therefore performed the last two immunizations with IDLV expressing a stabilized gp140 SOSIP Env trimer (CH505.w136 SOSIP) with the goal of focusing antibody responses towards the epitopes targeted by bnAbs. We observed that following IDLV-CH505.w136 SOSIP immunization there was the induction of Abs against epitopes only present on the closed Env trimer, while we did not detect a boost in the antibody response induced by the previous immunization with non-stabilized gp140 Envs.

Although we detected a narrower and more specific Ab response compared to immunizations with non-stabilized gp140 Envs, the two immunizations with IDLV-CH505.w136 SOSIP were not sufficient at inducing neutralization breadth. Recent studies demonstrated that there are several other factors working against the elicitation of bnAbs by vaccination, including host control, the paucity of bnAb precursors, the structure of the HIV-1 Env and the need for bnAbs to acquire improbable somatic mutations for their neutralization activity^28–30^. Substantial progress has been made in immunogen design to overcome some of those roadblocks. Saunders et al.^31^ recently demonstrated that a structurally modified CH505 Env could bind to the initial precursor of the bnAb lineage and could select for specific HIV-1 bnAb improbable mutations, resulting in the induction of potent neutralizing CD4 binding site Abs in macaques. Future studies will determine whether the expression of these novel immunogens by IDLV will drive bnAb maturation.

Although the induction of bnAbs by vaccination is desirable, results from human HIV-1 vaccine trials (RV144, APPROACH, and IPCAVD010/HPX1002) as well as from preclinical studies in NHPs suggest that non-neutralizing Abs could also reduce the risk of virus acquisition and mediate protection^3,32–35^. We therefore evaluated the efficacy of sequential IDLV-CH505 +/− protein vaccination against autologous virus acquisition. Only one animal in the IDLV-CH505 + protein co-immunization group resisted infection following 10 intrarectal challenges with SHIV.CH505.375H.dCT. This group of animals demonstrated 3.6 times lower peak viremia and better virus control compared to the control group of animals immunized with IDLV-GFP or IDLV-CH505 alone. Interestingly, we observed a positive correlation between the magnitude of binding and nAb responses and time to virus acquisition. Significant differences in binding to AVERY, C2, V2, and V5 epitopes were also observed between controller and non-controller animals. In addition, animals that required more than six challenges to become infected demonstrated stronger binding to AVERY and C5 epitopes. Altogether these data suggest that the differences in the magnitude of the antibody responses induced by the two vaccine regimens (IDLV-CH505 alone and IDLV-CH505 + protein) resulted in a more favorable challenge outcome in the co-immunization group.

Another important aspect we addressed in this study is the safety of the IDLV-vaccine following infection with a replication-competent virus. While there are a number of safety features built into the SIV-based IDLV used for immunization, testing these features in the setting of viral infection is critical, especially regarding the chance for a recombination event resulting in replication-competent virus. We have previously demonstrated that immunization of rhesus macaques with IDLV-Env did not result in the generation of RCLs in vivo^11^. Here, we assessed the safety of sequential IDLV vaccinations by evaluating vector mobilization and/or recombination events in PBMC and lymph nodes samples of 4 macaques vaccinated with sequential IDLV-CH505 and then challenged intravenously with SHIV.CH505. No evidence of vector mobilization or recombination between IDLV and the challenge virus could be detected.

In summary, the use of IDLV as a vaccine delivery system to express HIV-1 Env is a promising approach, particularly in light of its safety profile, the higher durability of immune responses, higher magnitude of neutralization and persistence of antigen expression compared to DNA and protein vaccination strategies. While IDLV proved successful at inducing high titer and durable immune responses, the immunogen strategy utilizing sequential non stabilized/optimized trimers did not induce tier 2 antibody responses, and this has been consistent across the different vaccine platforms used to deliver sequential gp120 or gp140 CH505 Envs. However, recent data from our group and others have demonstrated that conformationally stabilized Env trimer immunogens, in contrast to non-stabilized Envs, consistently induce autologous tier-2 nAbs^4,31,36^. Future studies will evaluate the ability of IDLV delivering stabilized Env trimers to enhance B-cell maturation and drive bnAb development.

## Methods

### Construction of IDLV-CH505 Envs plasmids

The clade C HIV-1 Env C.CH505 gp140 glycoproteins (T/F, w.53, w.78, w.100, and w.136 SOSIP)^5^ were cloned into an SIV-based SIN lentiviral transfer vector downstream of the internal CMV promoter (pGAE-CMV-CH505-Env-Wpre). The CH505.w.136 SOSIP trimer was generated as follow: the CH505 gp120 was grafted onto the BG505 gp41 forming a chimeric SOSIP^37^ with improved stability. The CH505 sequence was fused to BG505 sequence in the alpha 5 helix. We introduced E64K and A316W mutations^38^, of which have been shown to keep SOSIP trimers in the pre-CD4 bound state. The transfer vector pGAE-CMV-GFP-Wpre expressing the green fluorescent protein (GFP), the IN-defective packaging plasmids pAd-SIV-D64V, and the vesicular stomatitis virus Env glycoproteins (VSV-G) from the Indiana, New Jersey, and Cocal serotypes, used for vector pseudotyping, have been previously described ^12,25,39^.

### Vector production and validation

The human epithelium kidney 293T Lenti-X cells (Clontech Laboratories, Mountain View, CA) were maintained in Dulbecco’s Modified Eagles medium (Thermo Fisher Scientific, Waltham, MA) supplemented with 10% fetal bovine serum (GE Healthcare Life Sciences, HyClone Laboratories, South Logan, UT) and 100 units per ml of penicillin–streptomycin–glutamine (PSG) (Thermo Fisher Scientific). For the production of recombinant IDLV, 3.5 × 10^6^ Lenti-X cells were seeded on 100 mm diameter Petri dishes and transfected with 12 µg per plate of a plasmid mixture containing transfer vector, packaging plasmid and VSV.G plasmid in a 6:4:2 ratio, using the JetPrime transfection kit (Polyplus Transfection Illkirch, France) following the manufacturer’s recommendations. At 48 and 72 h post-transfection, culture supernatants were cleared from cellular debris by low-speed centrifugation and passed through a 0.45 μm pore size filter unit (Millipore, Billerica, MA). Filtered supernatants were concentrated by ultracentrifugation for 2 h at 71,000 × *g* on a 20% sucrose cushion. Pelleted vector particles were resuspended in 1× phosphate-buffered saline (PBS) and stored at −80 °C until further use. Each IDLV stock was titered using reverse transcriptase (RT) activity assay (RetroSys RT ELISA kit, Innovagen, Lund, Sweden) and the corresponding transducing units (TU) calculated by comparing the RT activity of each IDLV-CH505-Envs stock to the RT activity of IDLV-GFP stocks with known infectious titers^11^.

### Animals, IDLV-injection and SHIV challenge protocol

The thirty-one Indian origin rhesus macaques (*Macaca mulatta*) used in this study were negative for Mamu-A*01, Mamu-B*08, and Mamu-B*17 alleles and were housed at BIOQUAL, Inc. in accordance with the recommendations of the Association for Assessment and Accreditation of Laboratory Animal Care International Standards and with the recommendations in the Guide for the Care and Use of Laboratory Animals of the United States - National Institutes of Health. The Institutional Animal Use and Care Committee of BIOQUAL approved these experiments (study # 18-001).

Animals were immunized six times intramuscularly with sequential IDLV-CH505-Envs with 3 × 10^8^ TU per animal in 0.7-ml injection volume divided into two sites (left and right quadriceps). In the IDLV-CH505 + protein group, the animals received 100 μg of the corresponding CH505 gp140 protein in 25 μg of GLA-SE adjuvant (synthetic monophosphoryl lipid A in the stable emulsion)^19^ in addition to IDLV. Anticoagulated peripheral blood was obtained prior to IDLVs injection, 2 weeks post each immunization and monthly thereafter. Animals in groups B1, B2, and B3 were challenged ten times weekly by the intrarectal route with 1:100 dilution of SHIV.CH505.375H.dCT^17^ challenge stock (viral stock concentration = 178 ng/ml of p27Ag and 186,209 TCID50/mL as measured on rhesus CD4 + T cells). Animals in group A were challenged once intravenously with the same SHIV.CH505.375H.dCT dose at 6 weeks post the last immunization, as for animals in groups B1, B2 and B3. The virus stock was grown from the infectious molecular clone in rhesus PBMCs and the stock was titrated in rhesus macaques to select the appropriate dilution.

SHIV plasma viral RNA measurements were performed at the Immunology Virology Quality Assessment Center Laboratory Shared Resource, Duke Human Vaccine Institute, Durham, NC as described.

### Direct ELISAs

High binding EIA/RIA 384 well plates (Corning) were coated overnight with 2 µg ml^−1^ of CH505.T/F gp140 protein in coating buffer (KPL, Gaithersburg, MD). After 1 wash with washing buffer (1× PBS + 0.1% Tween 20) plates were treated for 1 h at room temperature with 40 μl per well of blocking buffer (PBS containing 4% [wt/vol] whey protein–15% normal goat serum–0.5% Tween 20). Serial threefold dilutions of plasma (from 1:3000 to 1:729,000) or monoclonal Abs (from 100 μg mL^−1^ to 0.5 ng mL^−1^) in blocking buffer were added to the plates (10 μl per well) in duplicates and incubated for 1.5 h at room temperature. The Rhesus B12 IgG (b12R1) was used to develop standard curves (range 100–0.005 ng mL^−1^, with each dilution assayed in duplicate). Abs were detected by adding 10 μl per well of horse radish peroxidase (HRP)-conjugated, polyclonal goat anti-monkey IgG (Rockland, Gilbertsville, PA, Cat # 617-103-012) diluted in blocking buffer (1:6000) and by adding 20 μl per well the SureBlue Reserve TMB microwell peroxidase substrate and stop solution (KPL, Gaithersburg, MD). Binding titers were analyzed as area-under-curve of the log transformed concentrations (Softmax Pro 7, Molecular Devices LLC, CA).

For detection of CH505.w136 SOSIP specific Abs in monkeys’ sera, PGT151 SOSIP capture ELISAs were performed as described^31^.

### Linear epitope mapping microarray

Specificity and magnitude of binding response to cross-subtype linear epitopes were measured in a peptide microarray assay^40^. The library contains overlapping peptides (15-mers overlapping by 12) covering 7 full-length gp160 consensus sequences (subtype A, B, C, D, Group M, CRF1, and CRF2 consensuses), gp120 sequences of six vaccine strains (MN, A244, Th023, TV-1, ZM641, 1086C), and sequential CH505 Env strains as gp120, gp140, and SOSIP sequences. Microarray scan images were analyzed using MagPix 8.0 software to obtain binding intensity values for all peptides. The magnitude of binding is calculated as the log2 fold difference, post-/pre-immunization intensity.

### HIV Neutralization assays

Neutralization of Env-pseudotyped viruses was measured in 96-well culture plates using Tat-regulated firefly luciferase (Luc) reporter gene expression to quantify reductions in virus infection in TZM-bl cells^41^. Serum neutralization was measured against: SVA-MLV (negative control for non-specific activity in the assay), CH505.w4.3 (tier 1), CH505.T/F (tier 2), CH505w53.16 (tier 2), CH505w.78 (tier 1b) and CH505w.100 (tier 2); SHIV CH505.375H (tier 2), CH505TF.gly3.276/GnTI- (tier 2), CH505TF.gly4/293S-GnTI- (tier 1b) and CH505s.G458Y.N279K/293S-GnTI- (tier 2). Heat-inactivated (56 °C, 1 h) serum samples were assayed at threefold dilutions starting at 1:20. Neutralization titers (50% inhibitory dose (ID50)) are the serum dilutions at which relative luminescence units (RLU) were reduced by 50% compared to RLU in virus control wells after subtraction of background RLU in cell control wells. A response was considered positive if the titer of the post-immunization ID50 was three times higher than that of the pre-immune ID50 and three times greater than the signal against the MLV-pseudotyped negative control virus.

### IFN-γ ELISpot assay

Multiscreen ninety-six well plates were coated overnight with 100 μl per well of 5 μg/ml anti-human interferon-γ (IFN-γ) (B27; Becton, Dickinson and Company, Franklin Lakes, NJ, Cat # 554699) in endotoxin-free Dulbecco’s-PBS (D-PBS). Plates were washed three times with D-PBS containing 0.1% Tween-20, blocked for 2 h with Roswell Park Memorial Institute 1640 medium (RPMI) containing 10% fetal bovine serum and incubated with peptide pools and 2 × 105 PBMCs in triplicate in 100 μl reaction volumes. Each peptide pool (1 μg/ml) was comprised of 15 amino acid peptides overlapping by 11 amino acids. The pools covered the entire HIV-Env and SIV-Gag proteins. Following 18-h incubation at 37 °C, plates were washed nine times with Dulbecco’s PBS containing 0.1% Tween-20 and once with distilled water. Plates were then incubated with 2 μg/ml biotinylated rabbit anti-human IFN-γ (U-CyTech biosciences, Utrecht, The Netherlands, Cat # CT243) for 2 h at room temperature, washed six times with D-PBS containing 0.1% Tween-20, and incubated for 1.5 hours with a 1:500 dilution of streptavidin-AP (Southern Biotechnology, Birmingham, AL, Cat# 7105-04). After five washes with D-PBS containing 0.1% Tween-20 and three washes with D-PBS alone, the plates were developed with bromochloroindolyl phosphate–nitro blue tetra-zolium (BCIP-NBT) chromogen (Thermo Fisher Scientific), stopped by washing with tap water, air dried, and read with an ELISpot reader (Cellular Technology Limited (CTL), Cleveland, OH) using ImmunoSpotAnalyzer software (Cellular Technology Limited (CTL)). Samples were considered positive if number of spot forming cells (SFC) was above twice that of the background (unstimulated) and >50 SFC per million PBMC.

### Vector and SHIV detection in PBMC and LNs

DNA was isolated from PBMC and lymph nodes cells collected 12 weeks post-challenge using the QIAamp micro kit (Qiagen) following the manufacturer’s instructions and eluted in 50 μL of water. Standard curves for LV-CH505 DNA were generated using serial dilutions of genomic DNA extracted from 293T cells transduced with an integrase competent lentiviral vector expressing the CH505 T/F gp140 Env (293T-LV-CH505). PCR reactions were performed using the primer sets and conditions shown in Supplementary Fig. 2. All alignments were made using gene cutter (hiv.lanl.gov) and phylogenetic trees were made with MEGA6^42^. Neighbor-joining trees were constructed using the Kimura 2-parameter mode and the reliability of topologies was estimated by performing bootstrap analysis with 1000 replicates.

### Statistical analysis

Comparisons between groups were made using exact-Wilcoxon tests because of the small sample size. Comparisons within groups across time points were made using Wilcoxon signed-ranked tests. An aligned rank test was used to compare nAb titers across time points. Correlations were assessed using Kendall’s Tau correlation test. No adjustment to the alpha level was made for multiple comparisons. All computations were made using SAS v9.4 (SAS Institute, Inc.).

### Reporting summary

Further information on experimental design is available in the Nature Research Reporting Summary linked to this paper.

## Supplementary information

Supplementary Information

Reporting Summary

## Data Availability

The data that support the findings of this study are available from the corresponding authors upon request.

## References

[1] Mascola JR, Haynes BF (2013). HIV-1 neutralizing antibodies: understanding nature’s pathways. Immunol. Rev..

[2] Williams WB (2015). HIV-1 VACCINES. Diversion of HIV-1 vaccine-induced immunity by gp41-microbiota cross-reactive antibodies. Science.

[3] Haynes BF (2012). Immune-correlates analysis of an HIV-1 vaccine efficacy trial. N. Engl. J. Med..

[4] Sanders RW (2015). HIV-1 VACCINES. HIV-1 neutralizing antibodies induced by native-like envelope trimers. Science.

[5] Liao HX (2013). Co-evolution of a broadly neutralizing HIV-1 antibody and founder virus. Nature.

[6] Doria-Rose NA (2014). Developmental pathway for potent V1V2-directed HIV-neutralizing antibodies. Nature.

[7] Gao F (2014). Cooperation of B cell lineages in induction of HIV-1-broadly neutralizing antibodies. Cell.

[8] Robb ML (2012). Risk behaviour and time as covariates for efficacy of the HIV vaccine regimen ALVAC-HIV (vCP1521) and AIDSVAX B/E: a post-hoc analysis of the Thai phase 3 efficacy trial RV 144. Lancet Infect. Dis..

[9] Rossi A (2014). Optimization of mucosal responses after intramuscular immunization with integrase defective lentiviral vector. PLoS ONE.

[10] Gallinaro A (2018). Integrase defective lentiviral vector as a vaccine platform for delivering influenza antigens. Front. Immunol..

[11] Negri D (2016). Immunization with an SIV-based IDLV expressing HIV-1 Env 1086 clade C elicits durable humoral and cellular responses in rhesus macaques. Mol. Ther..

[12] Blasi M (2018). IDLV-HIV-1 Env vaccination in non-human primates induces affinity maturation of antigen-specific memory B cells. Commun. Biol..

[13] Cousin C (2019). Persistence of integrase-deficient lentiviral vectors correlates with the induction of STING-independent CD8(+) T cell responses. Cell Rep..

[14] Lin YY (2020). Skeletal muscle is an antigen reservoir in integrase-defective lentiviral vector-induced long-term immunity. Mol. Ther. Methods Clin. Dev..

[15] Naldini L (1996). In vivo gene delivery and stable transduction of nondividing cells by a lentiviral vector. Science.

[16] Bonsignori M (2016). Maturation pathway from germline to broad HIV-1 neutralizer of a CD4-mimic antibody. Cell.

[17] Li H (2016). Envelope residue 375 substitutions in simian-human immunodeficiency viruses enhance CD4 binding and replication in rhesus macaques. Proc. Natl Acad. Sci. USA.

[18] Bar, K. J. et al. Simian-human immunodeficiency virus SHIV.CH505 infection of rhesus macaques results in persistent viral replication and induces intestinal immunopathology. *J. Virol.*10.1128/JVI.00372-19 (2019). 10.1128/JVI.00372-19 PMC6714786 31217249

[19] Coler RN (2011). Development and characterization of synthetic glucopyranosyl lipid adjuvant system as a vaccine adjuvant. PLoS ONE.

[20] Sanders RW (2013). A next-generation cleaved, soluble HIV-1 Env trimer, BG505 SOSIP.664 gp140, expresses multiple epitopes for broadly neutralizing but not non-neutralizing antibodies. PLoS Pathog..

[21] Sanders RW, Moore JP (2017). Native-like Env trimers as a platform for HIV-1 vaccine design. Immunol. Rev..

[22] LaBranche CC (2019). Neutralization-guided design of HIV-1 envelope trimers with high affinity for the unmutated common ancestor of CH235 lineage CD4bs broadly neutralizing antibodies. PLoS Pathog..

[23] Williams WB (2017). Initiation of HIV neutralizing B cell lineages with sequential envelope immunizations. Nat. Commun..

[24] Saunders KO (2017). Vaccine induction of heterologous tier 2 HIV-1 neutralizing antibodies in animal models. Cell Rep..

[25] Blasi M (2020). Therapeutic vaccination with IDLV-SIV-Gag results in durable viremia control in chronically SHIV-infected macaques. NPJ Vaccines.

[26] Julien JP (2015). Design and structure of two HIV-1 clade C SOSIP.664 trimers that increase the arsenal of native-like Env immunogens. Proc. Natl Acad. Sci. USA.

[27] Ringe, R. P. et al. Reducing V3 antigenicity and immunogenicity on soluble, native-like HIV-1 Env SOSIP trimers. *J. Virol.*10.1128/JVI.00677-17 (2017). 10.1128/JVI.00677-17 PMC5512241 28539451

[28] Wiehe K (2018). Functional relevance of improbable antibody mutations for HIV broadly neutralizing antibody development. Cell Host Microbe.

[29] Haynes, B. F., Burton, D. R. & Mascola, J. R. Multiple roles for HIV broadly neutralizing antibodies. *Sci. Transl. Med.*10.1126/scitranslmed.aaz2686 (2019). 10.1126/scitranslmed.aaz2686 PMC7171597 31666399

[30] Kwong PD, Mascola JR (2018). HIV-1 vaccines based on antibody identification, B cell ontogeny, and epitope structure. Immunity.

[31] Saunders, K. O. et al. Targeted selection of HIV-specific antibody mutations by engineering B cell maturation. *Science*10.1126/science.aay7199 (2019). 10.1126/science.aay7199 PMC7168753 31806786

[32] Barouch DH (2018). Evaluation of a mosaic HIV-1 vaccine in a multicentre, randomised, double-blind, placebo-controlled, phase 1/2a clinical trial (APPROACH) and in rhesus monkeys (NHP 13-19). Lancet.

[33] Stephenson, K. E. et al. Comparison of shortened mosaic HIV-1 vaccine schedules: a randomised, double-blind, placebo-controlled phase 1 trial (IPCAVD010/HPX1002) and a preclinical study in rhesus monkeys (NHP 17-22). *Lancet HIV*, 10.1016/S2352-3018(20)30001-1 (2020). 10.1016/S2352-3018(20)30001-1 PMC7297076 32078815

[34] Blasi, M. & Fouda, G. G. Shortening HIV vaccine regimens to achieve high coverage. *Lancet HIV*, 10.1016/S2352-3018(20)30039-4 (2020). 10.1016/S2352-3018(20)30039-4 PMC7276282 32078816

[35] Felber BK (2020). Co-immunization of DNA and protein in the same anatomical sites induces superior protective immune responses against SHIV challenge. Cell Rep..

[36] Steichen, J. M. et al. A generalized HIV vaccine design strategy for priming of broadly neutralizing antibody responses. *Science*10.1126/science.aax4380 (2019). 10.1126/science.aax4380 PMC7092357 31672916

[37] Zhou T (2017). Quantification of the impact of the HIV-1-glycan shield on antibody elicitation. Cell Rep..

[38] de Taeye SW (2015). Immunogenicity of stabilized HIV-1 envelope trimers with reduced exposure of non-neutralizing epitopes. Cell.

[39] Trobridge GD (2010). Cocal-pseudotyped lentiviral vectors resist inactivation by human serum and efficiently transduce primate hematopoietic repopulating cells. Mol. Ther..

[40] Shen X (2015). Vaccine-induced linear epitope-specific antibodies to Simian immunodeficiency virus SIVmac239 envelope are distinct from those induced to the human immunodeficiency virus type 1 envelope in nonhuman primates. J. Virol..

[41] Montefiori DC (2009). Measuring HIV neutralization in a luciferase reporter gene assay. Methods Mol. Biol..

[42] Tamura K, Stecher G, Peterson D, Filipski A, Kumar S (2013). MEGA6: molecular evolutionary genetics analysis version 6.0. Mol. Biol. Evol..

