# Supplementary material for: Immunogenicity, safety, and efficacy of sequential immunizations with an SIV-based IDLV expressing CH505 Envs
Source: NPJ Vaccines. 2020 Nov 18;5:107. doi: 10.1038/s41541-020-00252-w (PMC7674457; doi:10.1038/s41541-020-00252-w)
Supplement: Supplementary file 1 — Supplementary Information [file 41541_2020_252_MOESM1_ESM.pdf]

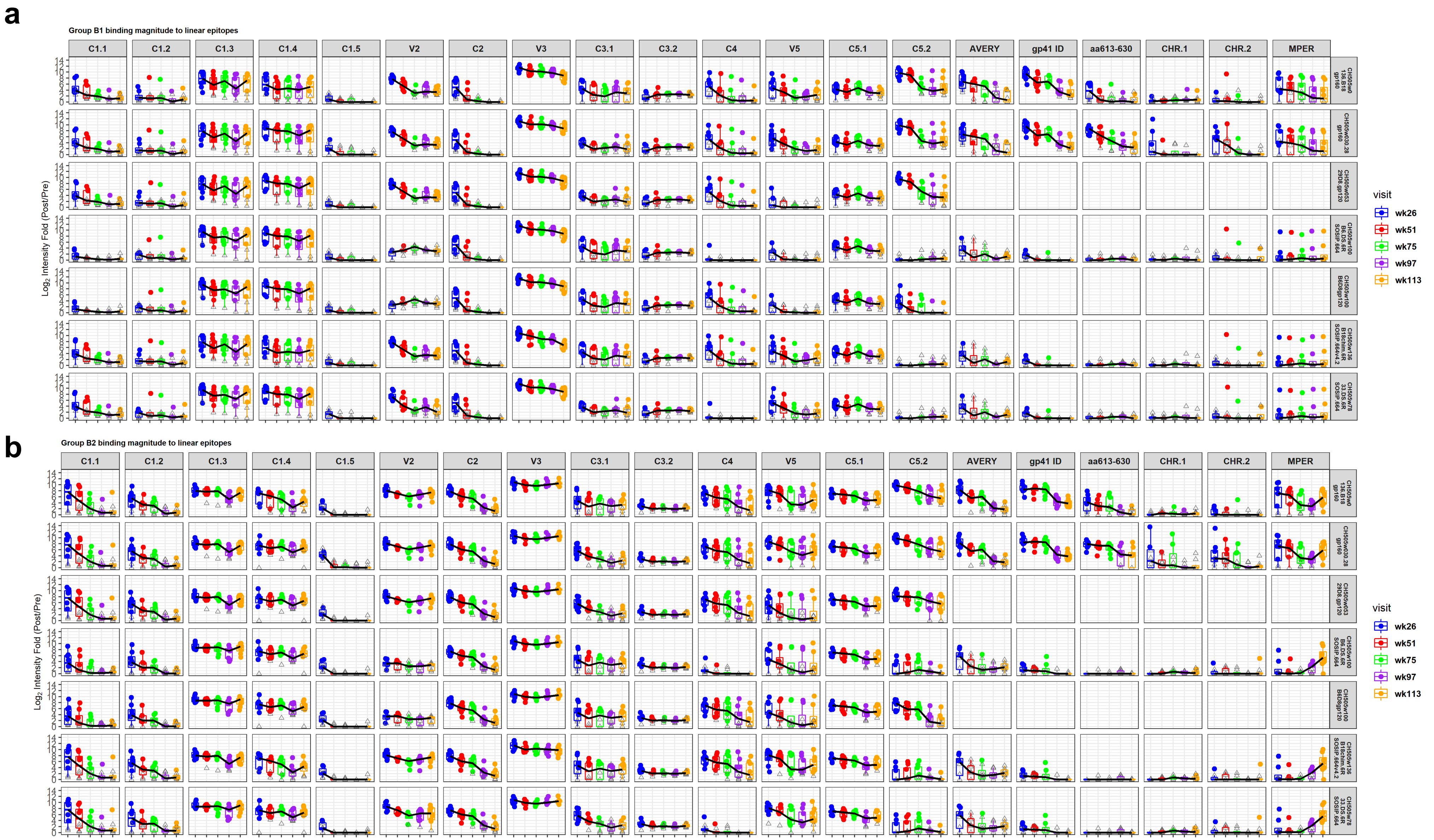

Supplementary Figure 1. Linear epitope binding to CH505 sequences in IDLV-CH505 +/- protein sequentially vaccinated animals. Linear epitope specificities at 2 weeks after each immunization are shown for both IDLV-CH505 alone (**a**) and IDLV-CH505 + protein (**b**) immunized animals. Each color indicates a different time point. Binding intensity is shown for each peptide, corrected with its own background value.

		Wk 113	wk 134 (wk 17 post-challenge)		
Vaccine Group	Animal ID	CH505.w4.3	CH505.w4.3	CH505 T/F	25710-2.43
**Group B1 IDLV-CH505 alone**	6597	<20	19693	41	128
	6599	82	13798	<20	24
	6595	21	23491	22	22
	6575	64	3676	91	<20
	6592	44	708	<20	<20
	6596	63	10996	84	30
	6586	336	8327	<20	26
	6589	34	1922	<20	<20
**Group B2 IDLV-CH505 + protein**	6582	2030	6492	40	<20
	6600	587	12146	<20	27
	6601	1095	763	<20	24
	6578	32	3709	37	<20
	6603	897	5128	<20	26
	6598	297	2110	<20	25
	6577	76	1551	<20	<20
	6584	87	1431	<20	<20

ID50
<20
20-100
100-1000
1000-10,000
>10,000

Supplementary Table 1. Serum neutralization activity against the clade C tier1 virus CH505.w4.3 and the clade C tier 2 viruses CH505 T/F and 25710-2.43 at wk 113 (4 weeks before challenge) and week 134 (17 weeks post-challenge).

a

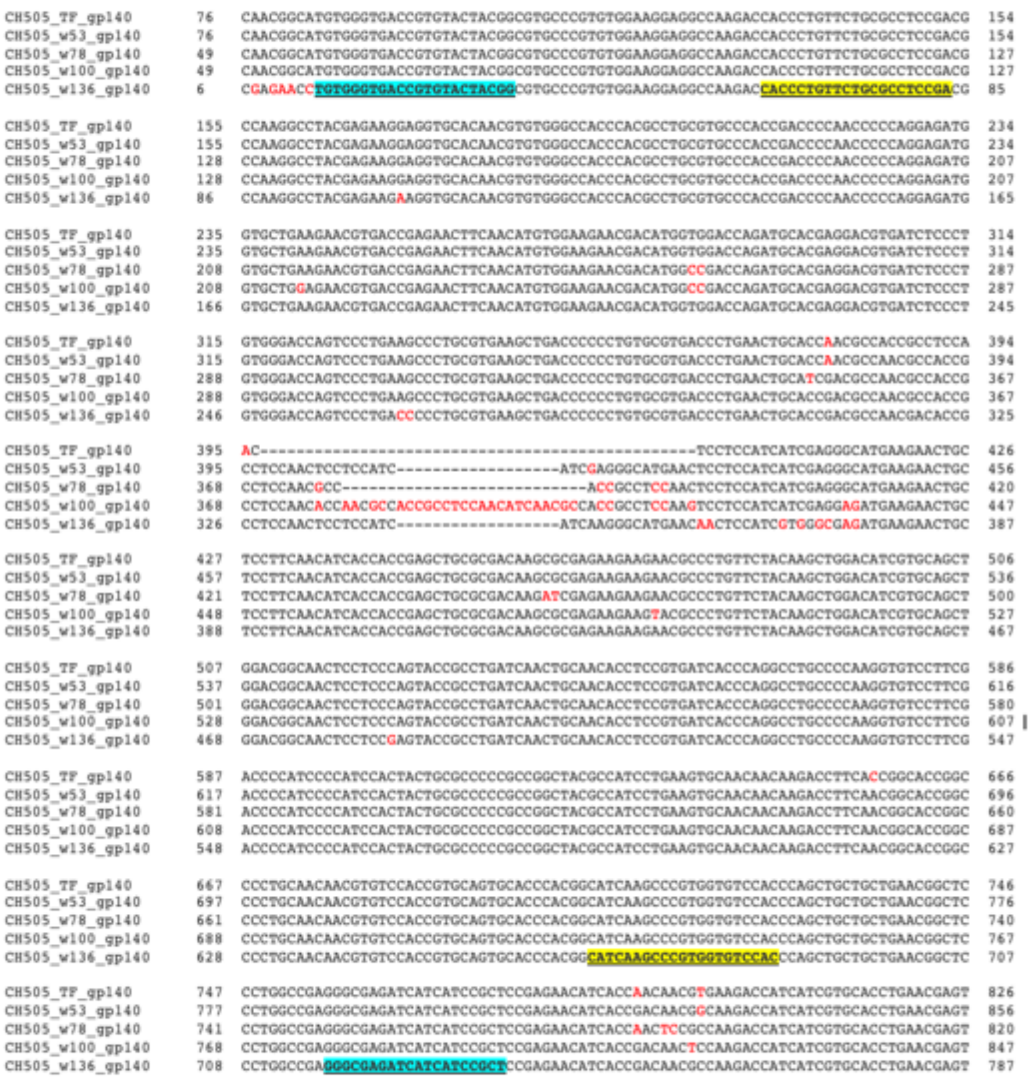

b

Primers and PCR conditions for the detection of IDLV and SHIV

Target	Primer	Sequence (5'-3')	PCR conditions
**SIV-IDLV-CH505**			
Outer	CH505_Env5'_Ex<br>CH505_Env3'_Ex	TGT GGG TGA CCG TGT ACT ACG G<br>AGC GGA TGA TGA TCT CGC CC	94°C 15s - 59°C 30s - 68°C 1 min for 16 cycles
Nested	CH505_Env5'_In<br>CH505_Env3'_In	CAC CCT GTT CTG CGC CTC CG<br>GTG GAC ACC ACG GGC TTG ATG	94°C 15s - 59°C 30s - 68°C 1 min for 31 cycles
**SHIV-CH505**			
Outer	SIVmac.F4out<br>SHIVEnvR3out	TCA TAT CTA TAA TAG ACA TGG AGA CAC CC<br>CTA ATT CCT GGT CCT GAG GTG TAA TCC TG	94°C 15s - 58°C 30s -68°C 4 min for 35 cycles
Nested	SIVmac766.F2in<br>SIVmac766.R2in	GGA AAT CCT CTC TCA ACT ATA CCG CCC TC<br>CTA TTG CCA ATT TGT AAC TCA TTG TTC	94°C 15s - 58°C 30s -68°C 4 min for 45 cycles

c

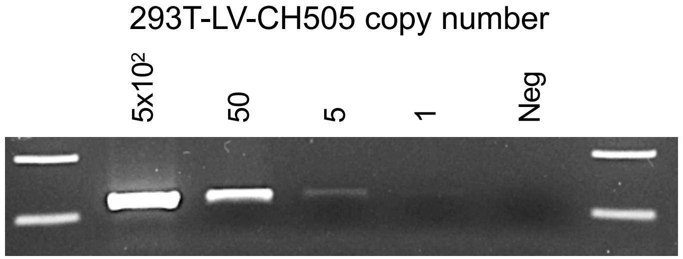

Supplementary Figure 2. PCR primers and conditions used to detect possible mobilization and or recombination events between IDLV-CH505 and SHIV-CH505 following intravenous challenge. (a) Alignment of the CH505 sequences encoded by the IDLV-CH505 vaccines used in this study. Primer sequences are highlighted in blue for the outer primers and in yellow for the inner primers. (b) PCR primers and conditions used to detect IDLV-CH505 or SHIV-CH505 sequences. (c) Standard curves for LV-CH505 DNA were generated using serial dilutions of genomic DNA extracted from 293T cells transduced with an integrase competent lentiviral vector expressing the CH505 T/F gp140 envelope (293T-LV-CH505). Neg = genomic DNA from non transduced cells.
